# Predicting Atrial Fibrillation Relapse Using Bayesian Networks: Explainable AI Approach

**DOI:** 10.2196/59380

**Published:** 2025-02-11

**Authors:** João Miguel Alves, Daniel Matos, Tiago Martins, Diogo Cavaco, Pedro Carmo, Pedro Galvão, Francisco Moscoso Costa, Francisco Morgado, António Miguel Ferreira, Pedro Freitas, Cláudia Camila Dias, Pedro Pereira Rodrigues, Pedro Adragão

**Affiliations:** 1 Department of Community Medicine, Information and Health Decision Sciences, Faculty of Medicine, University of Porto, Rua Dr Plácido da Costa, Porto, 4200-450, Portugal, 351 22 551 3622; 2 CINTESIS @ RISE – Center for Health Technology and Services Research, Porto, Portugal; 3 Cardiology and Electrophysiology Department, Hospital de Santa Cruz, Centro Hospitalar de Lisboa Ocidental, Carnaxide, Portugal

**Keywords:** artificial intelligence, atrial fibrillation, Bayesian networks, clinical decision-making, machine learning, prognostic models

## Abstract

**Background:**

Atrial fibrillation (AF) is a prevalent arrhythmia associated with significant morbidity and mortality. Despite advancements in ablation techniques, predicting recurrence of AF remains a challenge, necessitating reliable models to identify patients at risk of relapse. Traditional scoring systems often lack applicability in diverse clinical settings and may not incorporate the latest evidence-based factors influencing AF outcomes. This study aims to develop an explainable artificial intelligence model using Bayesian networks to predict AF relapse postablation, leveraging on easily obtainable clinical variables.

**Objective:**

This study aims to investigate the effectiveness of Bayesian networks as a predictive tool for AF relapse following a percutaneous pulmonary vein isolation (PVI) procedure. The objectives include evaluating the model’s performance using various clinical predictors, assessing its adaptability to incorporate new risk factors, and determining its potential to enhance clinical decision-making in the management of AF.

**Methods:**

This study analyzed data from 480 patients with symptomatic drug-refractory AF who underwent percutaneous PVI. To predict AF relapse following the procedure, an explainable artificial intelligence model based on Bayesian networks was developed. The model used a variable number of clinical predictors, including age, sex, smoking status, preablation AF type, left atrial volume, epicardial fat, obstructive sleep apnea, and BMI. The predictive performance of the model was evaluated using the area under the receiver operating characteristic curve (AUC-ROC) metrics across different configurations of predictors (5, 6, and 7 variables). Validation was conducted through four distinct sampling techniques to ensure robustness and reliability of the predictions.

**Results:**

The Bayesian network model demonstrated promising predictive performance for AF relapse. Using 5 predictors (age, sex, smoking, preablation AF type, and obstructive sleep apnea), the model achieved an AUC-ROC of 0.661 (95% CI 0.603‐0.718). Incorporating additional predictors improved performance, with a 6-predictor model (adding BMI) achieving an AUC-ROC of 0.703 (95% CI 0.652‐0.753) and a 7-predictor model (adding left atrial volume and epicardial fat) achieving an AUC-ROC of 0.752 (95% CI 0.701‐0.800). These results indicate that the model can effectively estimate the risk of AF relapse using readily available clinical variables. Notably, the model maintained acceptable diagnostic accuracy even in scenarios where some predictive features were missing, highlighting its adaptability and potential use in real-world clinical settings.

**Conclusions:**

The developed Bayesian network model provides a reliable and interpretable tool for predicting AF relapse in patients undergoing percutaneous PVI. By using easily accessible clinical variables, presenting acceptable diagnostic accuracy, and showing adaptability to incorporate new medical knowledge over time, the model demonstrates a flexibility and robustness that makes it suitable for real-world clinical scenarios.

## Introduction

Atrial fibrillation (AF), the most common sustained cardiac arrhythmia [1], poses significant challenges in the clinical management and prediction of disease progression. Currently, the ATLAS score [2] provides a reliable risk estimate to predict the rate of AF recurrence after a pulmonary vein isolation (PVI) procedure. However, it suffers from typical limitations of clinical scores, such as the use of a fixed number of independent variables for the prediction of a single dependent variable, its static nature, and its inability to be adjusted as new knowledge becomes available. All these issues can be addressed by artificial intelligence (AI) models based on machine learning algorithms, which can learn from available data, be quickly updated with new data, and perform complex calculations in a short time.

In recent years, such machine learning techniques have emerged as powerful tools in various medical domains, including cardiology [3,4]. There have been some recent successful attempts to develop AI models to predict the recurrence of AF after ablation procedure. However, despite the good performance of those models, they either lack the explainability required to allow their acceptance by health care professionals [5,6], or share the same limitations of medical scores discussed above [7]. In fact, although many physicians have recognized that AI models may be useful both for diagnosis and prognosis in medical practice, many authors raise legitimate questions about the lack of explainability of some AI models [8,9].

Bayesian networks, despite being still poorly adopted in health care, have gained popularity as clinical decision support models in medicine due to their ability to handle complex problems with causal dependencies, integrate both data and domain knowledge, provide an interpretable graphical structure, and support both diagnostic and prognostic reasoning [10]. In addition, these models can be updated with new medical knowledge, enabling the incorporation of novel risk factors and advancements in the field of arrhythmology. This adaptability and scalability make Bayesian networks a promising tool for decision-making in medicine and long-term monitoring of patients with AF.

This study aims to address key research gaps in the prediction of AF relapse by developing a more reliable and adaptable predictive model based on Bayesian networks. Traditional medical scoring systems are limited by their reliance on a fixed set of independent variables, which reduces their generalizability across diverse patient populations. In addition, many existing AI models for AF prediction lack the necessary explainability required to foster trust and acceptance among health care professionals. To bridge these gaps, this study makes several significant contributions. First, it introduces a novel explainable AI model based on Bayesian networks, which allows for the calculation of conditional probabilities tailored to individual patient profiles, thus enhancing both the interpretability of the predictions and their clinical acceptance. Second, the study overcomes the limitations of traditional scoring systems by offering a dynamic and adaptable model that can incorporate new risk factors and learn from evolving patient data, thereby improving predictive accuracy over time. Third, the proposed model demonstrates flexibility and robustness, making it suitable for real-world clinical scenarios where incomplete data may be present. Finally, by integrating this model into clinical decision support systems, the study has the potential to enhance decision-making processes and improve patient outcomes in the management of AF. In this work, we investigate the use of Bayesian networks to predict AF relapse before a percutaneous PVI procedure and evaluate its potential as a valuable clinical tool, with the primary aim of improving clinical decision-making and patient care.

## Methods

### Study Population

All consecutive patients with symptomatic drug-refractory AF undergoing cardiac computed tomography (CT) before percutaneous PVI at Hospital Santa Cruz (Carnaxide, Portugal) between November 2015 and July 2019 were included in an observational registry used for this retrospective study. Patients with moderate or severe valvular heart disease, left atrial thrombus, abnormal thyroid function, or contraindication to anticoagulation were excluded. Baseline demographic and clinical characteristics, including age, sex, height, weight, and presence of hypertension, diabetes, smoking, and known coronary artery disease, were recorded for all patients. AF was categorized as paroxysmal if it self-terminated in less than 7 days, persistent if episodes lasted ≥7 days or required cardioversion, or long-standing persistent if AF was maintained for more than 12 months.

### PVI Protocol

PVI was guided by electroanatomical mapping, using either NavX (St Jude Medical) or CARTO (Biosense Webster) systems. The right femoral vein was used as the preferred vascular access, through which three catheter electrodes were introduced: (1) a decapolar catheter, advanced through the coronary sinus; (2) a variable circular mapping catheter, placed in the pulmonary veins (PVs); and (3) an irrigated contact force-sensing ablation catheter. Left atrial access was established by a transseptal puncture. Radiofrequency ablation was performed more than 5 mm from the PV ostia, with continuous lesions enclosing the left and right pairs of PVs. The treatment was considered successful if complete electrophysiological PVI was achieved. When required, electrical cardioversion was performed at the end of the procedure. Oral anticoagulation was resumed 6 hours after the ablation, maintained for 6 months, and then withdrawn or continued according to CHA2DS2-VASc criteria. Generally, class I/III antiarrhythmic drugs were maintained in all patients for the first 3 months after the procedure and then withdrawn if there was no AF recurrence. A proton pump inhibitor was also prescribed for the first month after the ablation.

### Study End Point and Patient Follow-Up

The study end point was AF recurrence, defined as symptomatic or documented AF or other atrial arrhythmias, after a 3-month blanking period. Symptomatic AF was defined as the presence of symptoms considered to be likely due to AF episodes. Documented AF was defined by the presence of at least one episode of AF lasting more than 30 seconds in an ECG, 24-hour Holter monitoring, or event-loop recording. The follow-up protocol comprised outpatient visits with 12-lead ECG and 24-hour Holter monitoring at the assistant physicians’ discretion (typically at 6 and 12 months, and yearly thereafter). Patients were encouraged to contact the department if they experienced symptoms of AF recurrence. Whenever clinical records were insufficient, a structured telephonic interview was conducted. Patients who were kept on antiarrhythmic drugs after the third month of follow-up were not considered as failed ablation.

### Population Characteristics

The analyzed sample comprised demographic and clinical data from 480 patients who underwent follow-up after the PVI procedure described above. The cohort included 295 (61.5%) men and 185 (38.5%) women, with a mean age of 61.1 (SD 11.5) years. The median duration of the follow-up time of the patients was 392 (IQR 150‐674) days. For the purpose of this study, all numeric variables in the dataset (including age, BMI, left atrial volume, and epicardial fat) were discretized into classes. Data characterization is shown in Table 1.

**Table 1.** Demographic and clinical characteristics of the patients included in the study.

Characteristics	Total (N=480), n (%)	AF^a^ relapse (n=166), n (%)	AF-free (n=314), n (%)
**Sex**			
Female	185 (38.5)	55 (33.1)	130 (41.4)
Male	295 (61.5)	111 (66.9)	184 (58.6)
**Age (years)**			
≤45	57 (11.9)	9 (5.4)	48 (15.3)
46‐65	234 (48.8)	84 (50.6)	150 (47.8)
+65	189 (39.4)	73 (44)	116 (36.9)
Alcoholism	25 (5.2)	15 (9)	10 (3.2)
Smoking	135 (28.1)	57 (34.3)	78 (24.8)
Diabetes	46 (9.6)	16 (9.6)	30 (9.6)
High blood pressure	292 (60.8)	105 (63.3)	187 (59.6)
Obstructive sleep apnea	50 (10.4)	35 (21.1)	15 (4.8)
**BMI**			
Normal weight	151 (31.5)	35 (21.1)	116 (36.9)
Overweight	218 (45.4)	74 (44.6)	144 (45.9)
Obese	111 (23.1)	57 (34.3)	54 (17.2)
**Atrial fibrillation**			
Paroxysmal	374 (77.9)	98 (59)	276 (87.9)
Persistent	106 (22.1)	68 (41)	38 (12.1)
**Left atrium volume^b^ (ml/m^2^)**			
[0 to 100]	168 (35)	39 (23.5)	129 (41.1)
(100 to 125]	172 (35.8)	56 (33.7)	116 (36.9)
(125 to inf)	140 (29.2)	71 (42.8)	69 (22)
**Epicardial fat^b^ (cm^3^)**			
[0 to 2.7]	162 (33.8)	18 (10.8)	144 (45.9)
(2.7 to 4.6]	166 (34.6)	48 (28.9)	118 (37.6)
(4.6 to inf)	152 (31.7)	100 (60.2)	52 (16.6)

a AF: atrial fibrillation.

b Square brackets indicate that the end point is included in the range, and parentheses indicate that the end point is not included in the range.

The variable preablation AF type represents the type of AF identified in each patient before the ablation procedure, being coded either as paroxysmal or persistent. The variable sex is categorized as binary (female or male). All other binary variables such as alcoholism, smoking, diabetes, high blood pressure, and obstructive sleep apnea, were coded as logical (true or false), indicating the presence or absence of that condition.

The variable AF relapse represents the identification of postprocedural AF relapse in patients during follow-up examinations, also coded as logical (true or false). It was targeted as the outcome variable for this study.

### Bayesian Network Model Training

#### Network Structure

Considering that Bayesian networks are probabilistic graphical models made to represent knowledge, we started by building our network structure primarily based on medical knowledge in this field. In a first step, we opted to include (whitelist) some of the most noteworthy known clinical relationships between features, such as (1) known risk factors for diseases expressed in the dataset, namely diabetes, high blood pressure (HBP), and obstructive sleep apnea (OSA); and (2) known predictive features of AF relapse, such as the ATLAS score features (age, sex, smoking, persistent AF and left atrial volume), as well as epicardial fat [11,12] and OSA [13,14], as suggested by recent medical literature.

In the second step, we explored additional potential relationships between features that could improve model fit and better explain the observed data through data-driven inference. To achieve this, we applied a score-based structure learning method, using the Bayesian Information Criterion (BIC) [15] as the scoring metric to be optimized. The optimization of the BIC score was performed using a hill-climbing algorithm [16]. This approach allowed us to learn the remaining structure of the network, resulting in a model that aligns with current medical knowledge while effectively capturing the relationships between the variables.

#### Model Fitting

After the network structure was defined, a model could be set to learn the conditional probabilities among all related features. The parameters of the Bayesian network were thus fit given the previously learned structure and the available data, by means of a Bayesian posterior estimator with a uniform before. With the model fitted in this fashion, it was now possible to use the model to compute the estimated probability that a given patient has AF relapse given her clinical characteristics, for example, the model can be asked “based on the available data, what is the probability that a patient has AF relapse knowing that she is female,+65 years old and non-smoking*.*” Further examples of computed conditional probabilities for AF relapse based on patients’ conditions are presented in the *Results* section.

### Model Validation

Model validation was executed by out-of-sample testing to assess the predictive performance of the model on unseen data, as follows: from the full dataset, a random sample was taken to be used as training data for the model. This sample was used to train a conditional probabilities model, as previously described. Following that, the remaining observations that were not included in the training set were used as a test set, upon which the model predictions were tested. For this testing step, we used the model to compute the conditional probability of AF relapse for each patient in the test set, and stored the prediction results for each tested observation. This process was cyclically repeated multiple times until each observation had been used for testing at least 30 times. Finally, the calculated probability of AF relapse for each patient was assumed to be the average of all estimated probabilities for that patient. We then compared the average predicted probability with the true observation of AF relapse for each patient, and measured the performance through the area under the receiver operating characteristic curve (AUC-ROC).

Regarding the sampling process at the beginning of each cycle, it is worth mentioning that the random samples for training the model were obtained through one of four different sampling processes: (1) bootstrapping, which on average uses 63.2% of the observations for training, or (2) hold-out, using fixed splitting ratios for the train and test of 80:20, (3) 90:10, and (4) 95:5, that is, with 80%, 90%, and 95% of the observations, respectively, being used for training the model, and the remaining proportion used for testing. With these processes, we aimed to assess the model’s ability to generalize for unknown data and achieve a good estimator for the generalization error.

This analysis was carried out using R (version 4.2.2; R Foundation for Statistical Computing) [17], with packages *bnlearn* [18] and *pROC* [19].

### Ethical Considerations

This study adheres to the ethical guidelines of the Declaration of Helsinki, including its later amendments. It has been approved by the Health Ethics Commission of the Western Lisbon Hospital Center, with the approval number 2117. All patients provided written informed consent before this study for both the procedure and the publication of any relevant data. Patient confidentiality was maintained by removing any personally identifiable information from all data used in this study and its supplementary materials.

## Results

### Bayesian Network Structure

**Figure 1.**
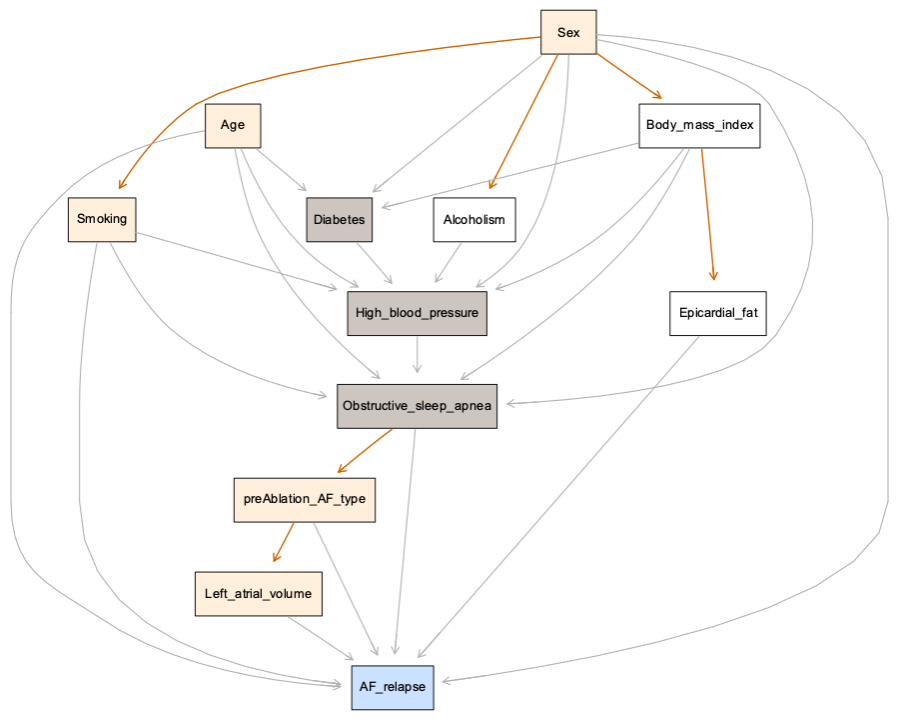
Bayesian network structure with nodes (boxes) representing the analyzed demographic and clinical variables. Grey nodes represent diseases with known associated risk factors, namely diabetes, high blood pressure, and obstructive sleep apnea. Beige nodes represent the 5 atrial fibrillation (AF) relapse predictors used by the ATLAS score, namely age, sex, smoking status, preablation AF type, and left atrial volume. The blue node highlights AF relapse as the outcome variable. The arcs (arrows) represent the direction of influence of variables. Grey arcs represent manually input relationships deriving from medical knowledge, ie, known risk factors. Orange colored arcs represent relationships discovered by the artificial intelligence algorithm, suggesting other meaningful relationships between variables.

The Bayesian network structure defined by expert knowledge and inference from data is represented in Figure 1.

As noted in this representation, the model suggests relationships that were not initially declared, such as BMI→Epicardial fat, OSA→preablation AF type, and preablation AF type→Left atrial volume. Furthermore, sex appears to be related to active smoking, alcoholism, and BMI. All these relationships are not surprising and are even supported by the current medical literature, thus providing a reasonable representation of clinical knowledge in this field. Regarding the outcome variable AF relapse, the model did not find any other relevant relations apart from those previously whitelisted.

**Figure 2.**
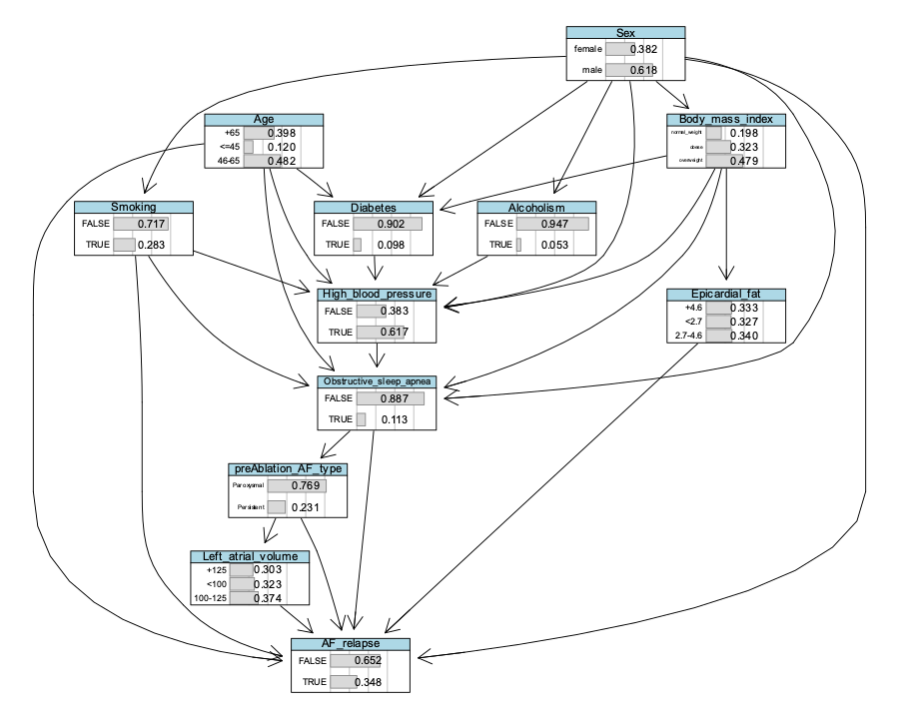
Bayesian network structure with node-specific tables displaying relative frequencies per class at each node. AF: atrial fibrillation.

An alternative representation of this network is exhibited in Figure 2, showing relative frequencies per class at each node.

### Conditional Probability Calculation

With each trained model, we calculated the conditional probability of AF relapse for each patient in the test set, considering their reported clinical conditions. These probabilities were compared with the true values of AF relapse for each patient and plotted in a receiver operating characteristic (ROC) curve, with cutoff values for classification determined as those that maximize the Youden J statistic. We tested in turns 7, 5, or 6 predictive features, as explained in the sections to follow. For illustration purposes, Table 2 presents a few examples of different combinations of patients’ conditions and their calculated conditional probability of AF relapse. These calculations were conducted for hypothetical patients, while considering as predictors all 7 parent nodes of AF relapse as represented in the network structure.

**Table 2.** Conditional probabilities of atrial fibrillation (AF) relapse for a sample of different combinations of hypothetical patients’ conditions. Conditions are sorted from the most unlikely to experience AF relapse to the most likely to experience that outcome.

Sex	Age (years)	Left atrium volume^a^ (ml/m^2^)	Smoking active	Persistent AF	Epicardial fat^a^ (cm^3^)	OSA^b^	Conditional probabilityof AF relapse, % (95% CI)
Male	≤45	[0 to 100]	False	Paroxysmal	[0 to 2.7]	False	7.5 (1.8-13.2)
Male	46‐65	(100 to 125]	False	Paroxysmal	[0 to 2.7]	False	10.1 (6.3-13.8)
Female	≤45	[0 to 100]	False	Paroxysmal	[0 to 2.7]	False	16.8 (7.4-26.1)
Male	46‐65	(125 to inf)	False	Paroxysmal	(2.7 to 4.6]	False	20.1 (14.3-26)
Male	+65	(100 to 125]	True	Paroxysmal	(2.7 to 4.6]	False	25.2 (17.3-33.1)
Male	46‐65	(100 to 125]	True	Persistent	[0 to 2.7]	False	33.2 (18.4-47.9)
Male	46‐65	(100 to 125]	False	Paroxysmal	(4.6 to inf)	True	33.3 (16.4-50.3)
Male	+65	(125 to inf)	False	Paroxysmal	(2.7 to 4.6]	False	33.3 (25.2-41.5)
Female	46‐65	[0 to 100]	False	Paroxysmal	(2.7 to 4.6]	False	40.1 (34-46.2)
Male	46‐65	[0 to 100]	True	Paroxysmal	(4.6 to inf)	False	50 (41.4-58.6)
Female	≤45	(100 to 125]	False	Paroxysmal	(4.6 to inf)	False	50.1 (35.7-64.5)
Male	46‐65	(100 to 125]	True	Paroxysmal	(4.6 to inf)	False	66.3 (57.4-75.1)
Female	+65	(125 to inf)	False	Persistent	(4.6 to inf)	False	66.4 (53.8-78.9)
Male	+65	(100 to 125]	False	Persistent	(4.6 to inf)	False	66.4 (52.6-80.2)
Female	46‐65	(100 to 125]	False	Paroxysmal	(4.6 to inf)	False	66.5 (59.9-73.1)
Male	+65	(125 to inf)	False	Paroxysmal	(4.6 to inf)	False	71.5 (63.8-79.2)
Male	+65	(125 to inf)	False	Persistent	(4.6 to inf)	False	74.8 (63.3-86.4)
Male	46‐65	(125 to inf)	True	Persistent	(4.6 to inf)	True	74.9 (58.4-91.4)

a Square brackets indicate that the end point is included in the range, and parentheses indicate that the end point is not included in the range.

b OSA: obstructive sleep apnea.

### The 7 Predictors

**Figure 3.**
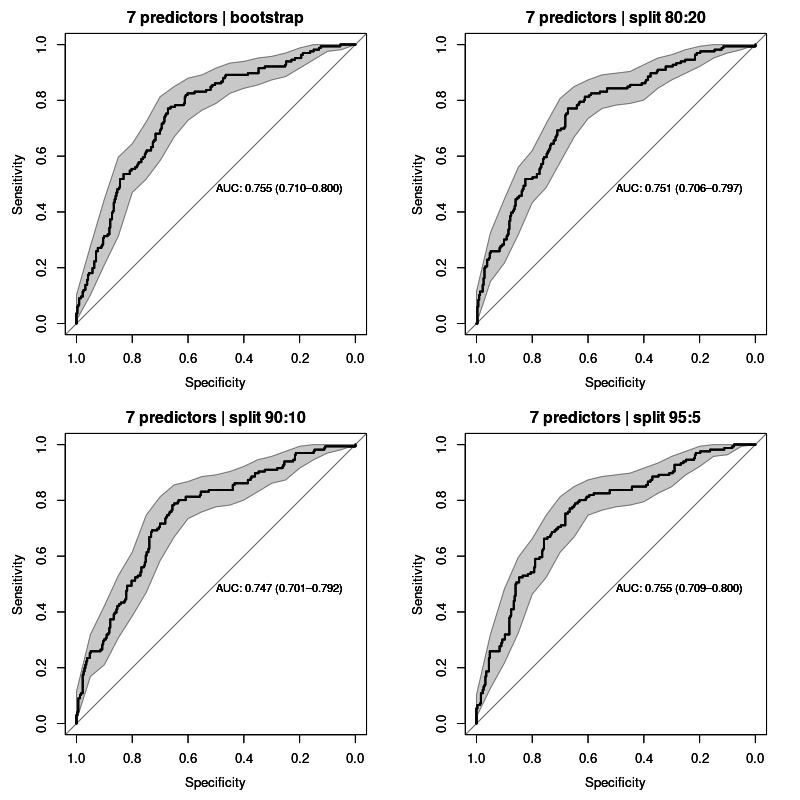
Receiver operating characteristic curves for all validation sampling methods applied to the model with 7 predictors: age, sex, smoking, preablation AF type, left atrial volume, epicardial fat, and obstructive sleep apnea. AUC values averaged 0.752 (95% CI 0.701‐0.800). AUC: area under the curve.

In the first stage, the calculation considered the clinical state of the patients for the 7 parent nodes of AF relapse represented in the network structure: age, sex, smoking, preablation AF type, left atrial volume, epicardial fat, and OSA. The performance of the model in classifying AF relapse with all parent nodes (7 predictors) was calculated to an average area under the curve (AUC) value of 0.752 (95% CI 0.701‐0.800) for all sampling methods. ROC curves for each validation test are shown in Figure 3.

### The 5 Predictors

Out of the 7 predictive features used in the previous test, 2 are usually difficult to obtain: left atrial volume and epicardial fat. These 2 features are typically calculated by diagnostic imaging, which is not always performed for all patients. In some cases, the physician does not have access to those measurements, which frustrates the calculation of medical scores that require any of those values, as is the case with the ATLAS score.

The purpose of this test was to evaluate the performance of the model without these 2 features, thus simulating a frequent real-life scenario. As such, we calculated the conditional probability of AF relapse for each patient in the test set, considering only 5 of its parent nodes: age, sex, smoking, preablation AF type, and OSA. The remaining 2 parent nodes (left atrial volume and epicardial fat) were disregarded from evidence to calculate conditional probabilities.

**Figure 4.**
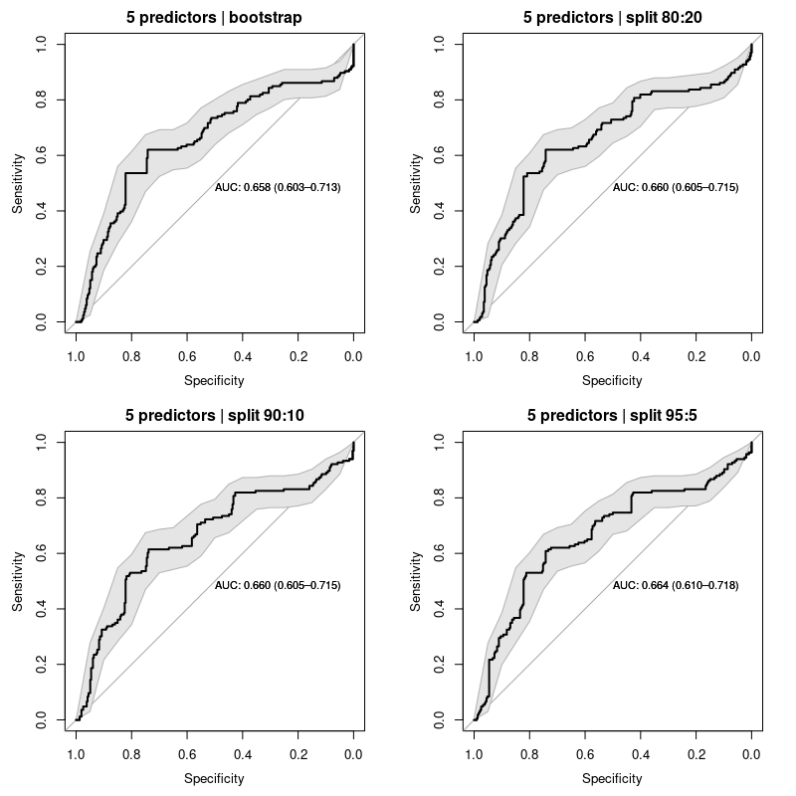
Receiver operating characteristic curves for all validation sampling methods applied to the model with 5 predictors: age, sex, smoking, preablation atrial fibrillation type, and obstructive sleep apnea. AUC values averaged 0.661 (95% CI 0.603‐0.718). AUC: area under the curve.

The performance of the model for classifying AF relapse with these 5 predictors was as expectably lower than with 7 predictors, with a calculated AUC average of 0.661 (95% CI 0.603‐0.718) for all sampling methods. ROC curves for each validation test are shown in Figure 4.

### The 6 Predictors

The predictive performance with only the previous 5 predictors appears to be slightly more than average. However, it can be observed from the defined Bayesian network structure (Figure 1) that the epicardial fat node has BMI as its single parent, meaning that the latter directly influences the former. As such, the lack of information on epicardial fat for a given patient can be partially compensated by its information on the BMI value. This poses an interesting possibility, especially when observed that BMI is usually an available or easy to obtain feature for any patient.

The rationale for this test was therefore to gauge the predictive power of a model when using the 5 predictors in the previous experience, plus the information on the BMI node. All these 6 features—age, sex, smoking, preablation AF type, OSA, and BMI—are usually easily available clinical variables for physicians’ evaluation, which do not require the use of additional complex or expensive diagnostic means. Therefore, this setting simulates the predictive power of the model in a likely real-life scenario.

For this test, we calculated the conditional probability of AF relapse for each patient in the test set, considering evidence on age, sex, smoking, preablation AF type, OSA, and BMI. Any information on left atrial volume and epicardial fat was ignored for this purpose.

**Figure 5.**
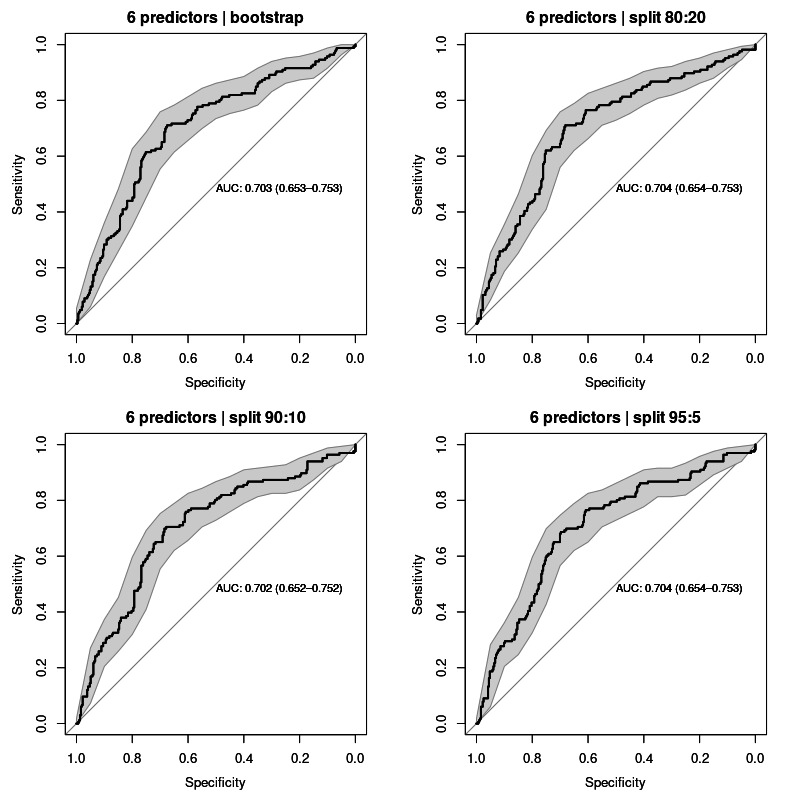
Receiver operating characteristic curves for all validation sampling methods applied to the model with 6 predictors: age, sex, smoking, preablation atrial fibrillation type, obstructive sleep apnea, and BMI. AUC values averaged 0.703 (95% CI 0.652‐0.753). AUC: area under the curve.

The performance of the model for classifying AF relapse with these 6 predictors resulted in a computed AUC average of 0.703 (95% CI 0.652‐0.753) for all sampling methods. ROC curves for each validation test are shown in Figure 5.

Table 3 presents a comparative analysis of the three models developed using 5, 6, and 7 predictors, respectively. As shown, the AUC-ROC progressively increases with the addition of predictors, indicating improved model performance. Furthermore, the 95% CI narrows as the number of predictors increases, suggesting greater precision in the model’s estimates.

**Table 3.** Comparative analysis of model performance based on the number of predictors and validation sampling techniques, using area under the receiver operating characteristic curve (AUC-ROC) metrics.

Model	AUC-ROC (95% CI)				
	Bootstrap	Split 80:20	Split 90:10	Split 95:5	Mean
5 predictors	0.658 (0.603‐0.713)	0.660 (0.605‐0.715)	0.660 (0.605‐0.715)	0.664 (0.610‐0.718)	0.661 (0.603‐0.718)
6 predictors	0.703 (0.653‐0.753)	0.704 (0.654‐0.753)	0.702 (0.652‐0.752)	0.704 (0.654‐0.753)	0.703 (0.652‐0.753)
7 predictors	0.755 (0.710‐0.800)	0.751 (0.706‐0.797)	0.747 (0.701‐0.792)	0.755 (0.709‐0.800)	0.752 (0.701‐0.800)

## Discussion

### Principal Findings

The ability to accurately predict clinical outcomes is vital for improving the quality of medical care and increasing the efficiency of resource allocation in health care. For such predictions, cardiologists often use clinical scores that have various limitations, such as being dependent on a set number of medical variables or not being adaptable to new medical knowledge. Nonetheless, these professionals have also been witnessing the development of AI models for applications in cardiology in general [20] and for the management of arrhythmias in particular [21,22]. In this context, our aim was to develop an alternative model to clinical scores that was not susceptible to these limitations, to predict the relapse of AF after PVI procedure.

For this purpose, we have resorted to Bayesian networks, a type of probabilistic graphical model that can represent knowledge as a set of variables and their conditional dependencies. Unlike traditional prognostic models based on linear or logistic regressions, Bayesian networks offer an interpretable graphical structure, which enhances the model’s clarity and facilitates its adoption among physicians. In addition, Bayesian networks manage missing data more efficiently than other machine learning methods like classification and regression trees or random forests, as they can compute the probability of an outcome even when predictive variables have missing values. This makes them particularly well suited for medical datasets, where missing data are often a challenge. We have therefore chosen to develop our models based on Bayesian networks due to their explainability, flexibility, and robustness. Their explainability derives from their ability to represent relationships between variables as a graphical model, thus rendering their results more comprehensible. This capability is of paramount importance for the acceptance of AI models by medical professionals, who can thus integrate them safely into clinical practice [23]. Further, the models’ flexibility derives from the ability to accommodate and represent new medical knowledge by reshaping the network structure accordingly and recalculating the conditional dependencies among multiple variables. Therefore, new suspected or known risk factors or predictors for AF relapse can be incorporated into a Bayesian network model at any time, with minimal resetting of the model. Additionally, the models’ robustness derives from the fact that they can make predictions for the outcome variable even when there are missing data on some predictive variables, thus allowing them to be used in cases of incomplete information on any given patient. Thus, unlike clinical scores, Bayesian networks do not require the full set of clinical explanatory variables to deliver useful results. Despite none of these characteristics being unique to Bayesian networks on its own, this combination of characteristics makes these models highly interesting to be used as basis for clinical decision support tools.The first stage of the construction of our model was to create the network structure, that is, the network of relationships between the clinical variables. As stated in the *Methods* section, this was achieved in 2 steps: initially the known relationships were set manually based on expert knowledge; then, in a second step, the network structure was improved upon inference from data by the use of an AI algorithm. At this last step, the algorithm suggested a relationship between BMI and epicardial fat, which was considered acceptable, as there is significant evidence of a correlation between these two variables [24]. This finding proved useful since it enabled the use of the path “BMI → epicardial fat → AF relapse” when there was no information on the middle variable. The algorithm also suggested a path “OSA → pre-ablation AF type → left atrial volume.” In this study, we opted to retain this suggestion in the network structure as a potential motivation for further exploration in future research. Although these relationships were considered to represent knowledge derived from the data, they were not particularly relevant for the model calculations, since each of these variables is also directly related to the outcome variable.

The second stage of the construction of our model was to train and validate the model based on the previous network structure. When validating the use of evidence from the 7 parent nodes of our outcome variable, the model performed with a calculated AUC value of approximately 0.75, interpreted as acceptable diagnostic accuracy [25]. These results implied using as predictive variables age, sex, smoking, preablation AF type, left atrial volume, epicardial fat, and OSA. However, some of these features are not always available in patients’ clinical records. Thus, we have validated the model in the absence of information on left atrial volume and epicardial fat as predictive features. In this case, the model exhibited an expectedly lower performance, with a calculated mean AUC value close to 0.66. Despite the observed difference was not statistically significant, as noted from the overlapping confidence intervals, it suggests that these 2 features have a high weight on the performance of the model. This finding is consistent with those reported in the ATLAS score that the left atrial volume has the highest weight on the predictive power of that score [2].

Going further, our experiment also showed that the lack of information on epicardial fat can be partially compensated for by evidence of BMI, as this is its parent node. Taking into account daily clinical practice, this poses an interesting possibility, since BMI measurements are generally available for clinical evaluation for most patients. In these 6-variable cases, the model response exhibited a calculated mean AUC value of 0.70. Also here, despite the observed differences for the previous scenarios not being statistically significant, these outcomes fit within an acceptable range for a prediction tool. Such results implied using as predictive variables age, sex, smoking, preablation AF type, OSA, and BMI, all of which are typically easy to obtain in a clinical setting. To put these results in perspective, the AFA Recur tool developed by Saglietto et al [5] achieves a performance of AUC 0.72 using a 19-variable AI model with little to no explainability.

Future research in the context of predicting AF relapse using Bayesian networks should address several key challenges and directions. The first is ensuring the generalizability of the model across diverse populations and clinical settings to seek validation in varied patient cohorts. Second, it would be essential to conduct longitudinal studies to assess the model’s long-term performance and capture patient evolution over extended time horizons. In addition, future studies could explore the inclusion of expanded predictive factors, such as genetic influences, lifestyle changes, and comorbidities, to enhance the model’s accuracy and clinical use. Finally, incorporating patient-reported outcomes and preferences into the predictive framework may improve the model’s relevance and acceptance, fostering a more patient-centric approach to clinical decision-making.

We consider that this data-based approach based on a Bayesian network model can be the backbone for a future clinical decision support system. Being an AI model, it opens the possibility of being continuously retrained as new patient information becomes available in clinical records, hence progressively providing more accurate results upon new accumulated data. Such a retraining process can be automatized on a schedule or upon a trigger, for example, recalculating conditional dependencies between clinical features on a monthly basis or at every new 100 patient observations. This retraining of the model based on the recalculation of conditional probabilities from new patient data is not expected to represent significant computational costs, even for exceptionally large amounts of patient observations.

This model can also be considered as an enhancement of the ATLAS score, as it is based on its 5 predictive features, to which 2 additional features were added. Nonetheless, it may serve as a starting point for the representation of knowledge in this field, being open to incorporating new evidence as it becomes available. For such a reason, we believe that the findings of this research contribute to the growing body of knowledge on the application of AI methods in cardiology and pave the way for future advancements in predictive analytics for cardiovascular diseases.

### Strengths and Limitations

The model was developed and evaluated on a dataset with a limited number of features. Although the current literature identifies other potential risk factors for relapse of AF, these were not considered in this work, as there was no information from patients on such features. Nevertheless, this type of model allows the incorporation of other risk factors at any time, provided that the network structure is rebuilt for that knowledge representation and the model is retrained accordingly.

In addition, the size of the dataset used in this work was below optimal for this type of probabilistic model. This is particularly relevant if we consider the subsample sizes for a given combination of clinical conditions (eg, in this dataset, there was only one observation that simultaneously satisfies the multiple conditions sex = female + smoking = true + OSA = true). However, this type of model can be set to learn from new patient data as they becomes available. In this fashion, as it continuously builds on new evidence, the model becomes more accurate and reliable, even for less frequent clinical conditions.

## Abbreviations

AF: atrial fibrillation
AI: artificial intelligence
AUC: area under the curve
AUC-ROC: area under the receiver operating characteristic curve
BIC: Bayesian Information Criterion
CT: computed tomography
HBP: high blood pressure
OSA: obstructive sleep apnea
PV: pulmonary vein
PVI: pulmonary vein isolation
ROC: receiver operating characteristic
